# Integrated Metagenomic and Transcriptomic Analyses Reveal the Dietary Dependent Recovery of Host Metabolism From Antibiotic Exposure

**DOI:** 10.3389/fcell.2021.680174

**Published:** 2021-06-18

**Authors:** Bingbing Li, Huihui Qiu, Ningning Zheng, Gaosong Wu, Yu Gu, Jing Zhong, Ying Hong, Junli Ma, Wen Zhou, Lili Sheng, Houkai Li

**Affiliations:** ^1^Institute of Interdisciplinary Integrative Medicine Research, Shanghai University of Traditional Chinese Medicine, Shanghai, China; ^2^Human Phenome Institute, Fudan University, Shanghai, China; ^3^Huzhou Key Laboratory of Molecular Medicine, Huzhou Central Hospital, Huzhou, China; ^4^Shanghai Veterinary Research Institute, Chinese Academy of Agricultural Sciences, Shanghai, China

**Keywords:** antibiotics, post-antibiotic recovery, diet, gut microbiota, metabolism

## Abstract

The balance of gut microbiome is essential for maintaining host metabolism homeostasis. Despite widespread antibiotic use, the potential long-term detrimental consequences of antibiotics for host health are getting more and more attention. However, it remains unclear whether diet affects the post-antibiotic recovery of gut microbiome and host metabolism. In this study, through metagenomic sequencing and hepatic transcriptome analysis, we investigated the divergent impacts of short-term vancomycin (Vac), or combination of ciprofloxacin and metronidazole (CM) treatment on gut microbiome and host metabolism, as well as their recovery extent from antibiotic exposure on chow diet (CD) and high-fat diet (HFD). Our results showed that short-term Vac intervention affected insulin signaling, while CM induced more functional changes in the microbiome. However, Vac-induced long-term (45 days) changes of species were more apparent when recovered on CD than HFD. The effects of antibiotic intervention on host metabolism were long-lasting, antibiotic-specific, and diet-dependent. The number of differentially expressed gene was doubled by Vac than CM, but was comparable after recovery on CD as revealed by the hepatic transcriptomic analysis. In contrast, HFD intake during recovery could worsen the extent of post-antibiotic recovery by altering infection, immunity, and cancer-related pathways in short-term Vac-exposed rats and by shifting endocrine system-associated pathways in CM-exposed rats. Together, the presented data demonstrated the long-term recovery extent after different antibiotic exposure was diet-related, highlighting the importance of dietary management during post-antibiotic recovery.

## Introduction

The human intestine is colonized by trillions of microbes that participate in many physiological and pathological processes of host including energy metabolism, xenobiotic metabolism, and immune response (Lozupone et al., 2012; Festi et al., 2014; Valdes et al., 2018). A stable and healthy gut microbiota structure is essential for maintaining metabolic homeostasis (Rinninella et al., 2019; Fan and Pedersen, 2021), preventing colonization by pathogens (Pickard et al., 2017; Ducarmon et al., 2019), improving immunity (Pickard et al., 2017), and extending lifespan (Biagi et al., 2016; Vaiserman et al., 2017). Mounting evidence in humans and rodents has shown that disruption of gut microbiota homeostasis and loss of bacterial diversity could cause metabolic disorders, neurological and immunological diseases, and impaired immunotherapy response (Gilbert et al., 2016; Gopalakrishnan et al., 2018a, b; Routy et al., 2018; Aron-Wisnewsky et al., 2020).

Many factors affect the composition of gut microbiota, such as antibiotic administration (Quigley, 2017). Antibiotics have been widely used to prevent and treat bacterial infections in humans and animals since the 1940s (Lewis, 2013; McPherson et al., 2018). However, exposure to antibiotics reduces the diversity of gut microbiota and leads to dysbiosis sometimes (Ianiro et al., 2016). There is increasing concern about the potential long-term detrimental consequences of antibiotic use for host health (Blaser, 2014; Cox and Blaser, 2015; Neuman et al., 2018). *Clostridium difficile* infection-associated diarrhea is a common consequence of antibiotic use (Guh and Kutty, 2018). Early life exposure to low-dose penicillin led to long-term increased adiposity, amplified diet-induced obesity (Cox et al., 2014), increased brain cytokine expression, and altered behavior in animal models (Leclercq et al., 2017). In addition, antibiotic-driven changes in microbiota affected glucose homeostasis and murine immune response and increased susceptibility to allergic asthma (Russell et al., 2012; Russell et al., 2013; Fujisaka et al., 2016). Epidemiological studies have found that early exposure to antibiotics is associated with subsequent development of obesity, inflammatory bowel diseases, allergic diseases, and detrimental neurodevelopmental outcomes (Hviid et al., 2011; Trasande et al., 2013; Forrest et al., 2017; Han et al., 2017; Hirsch et al., 2017; Slykerman et al., 2017; Mitre et al., 2018; Slykerman et al., 2019). These findings suggest that antibiotic-induced microbiome disruption can have long-term substantial effects on host health. However, how to eliminate the detrimental consequences of antibiotic use for host metabolism should particularly be given more attention.

Post-antibiotic recovery of gut microbiota composition is essential for the long-term health of host. Many studies have demonstrated that gut microbiota recovery following antibiotic treatment can be incomplete and the differential recovery of gut microbiota to the same antibiotic treatment is associated with their initial gut microbiota structure (Dethlefsen and Relman, 2011; Raymond et al., 2016a, b; Chng et al., 2020). For example, higher initial microbial diversity is positively correlated with better recovery from antibiotic-induced dysbiosis (Dethlefsen and Relman, 2011; Raymond et al., 2016a, b). In addition, certain bacterial species and enriched carbohydrate-degradation and energy-production pathways exhibit a robust association with post-antibiotic recovery (Chng et al., 2020). Nevertheless, besides the initial gut microbiota composition, our understanding on the factors that affect post-antibiotic recovery process is very limited. Diet is another critical factor influencing the composition of the gut microbiota within days (Carmody et al., 2015; Gentile and Weir, 2018; Kolodziejczyk et al., 2019; Zmora et al., 2019). Unbalanced diet was found to affect host health, such as causing metabolic disorders, immunological diseases, and neurological diseases (Brandsma et al., 2015; Gentile and Weir, 2018; Riccio and Rossano, 2018; Zmora et al., 2019; Tong et al., 2020). However, it is not clear whether and how environmental factors, such as diet, accelerate or impede the process of post-antibiotic recovery from different antibiotic-induced dysbiosis, which, in turn, affects the host phenotype and metabolism.

In this study, we investigated the divergent impacts of a 5-day intervention with two antibiotic regimens (vancomycin, Vac, or combination of ciprofloxacin and metronidazole, CM) on gut microbiota composition and host metabolism in rats, as well as the recovery from antibiotic exposure on either chow diet (CD) or high-fat diet (HFD) for 45 days. Our results revealed that the impacts of antibiotic exposure on host metabolism were long-lasting, antibiotic-specific, and diet-dependent. Compared to the effect of CD on post-Vac recovery, HFD affected post-Vac recovery of host metabolism and significantly regulated infection, immunity, and cancer-related pathways. In sum, diet plays a critical role in the recovery of host metabolism after antibiotic intervention, which points to the fact that the dietary management should particularly be given more attention at the post-antibiotic recovery stage.

## Materials and Methods

### Antibiotic Intervention Experiment

Male SD rats of 200-g body weight (BW) were provided by the Laboratory Animal Center of Shanghai University of Traditional Chinese Medicine (Shanghai, China). Rats were orally administrated with vehicle or vancomycin (100 mg/kg per dose) or combination of ciprofloxacin (50 mg/kg per dose) and metronidazole (50 mg/kg per dose) twice daily for 5 days. The dose was doubled at the first and last administration. For the insulin intervention experiment, 8 min after insulin injection (10 U/kg), rats were euthanized and tissues were collected for protein analysis (Stahel et al., 2017). For the recovery experiment, rats were fed with chow diet or high-fat diet for 45 days after antibiotic intervention. All rats were housed in a 12-h light (7 am to 7 pm) and 12-h dark (7 pm to 7 am) cycle, with free access to water and diet. The experiments were conducted under the Guidelines for Animal Experiment of Shanghai University of Traditional Chinese Medicine, and the protocol was approved by the institutional Animal Ethics Committee.

### Glucose and GTTs

Glucose-tolerance tests were performed on fasted rats (15 h, paper bedding) by monitoring glucose levels after a glucose bolus (1 g/kg of BW) by intraperitoneal injection (IP). Data acquisition was carried out at 0, 15, 30, 60, 90, and 120 min after injection. For the diet stimulation experiment, blood glucose was measured on fasted rats (15 h) and re-fed rats (2 h) (Dick et al., 2015).

### Biochemical Analysis

Serum biochemical indices such as triglycerides (TG), cholesterol (TC), alanine aminotransferase (ALT), aspartate aminotransferase (AST), low-density lipoprotein (LDL), and high-density lipoprotein (HDL) were determined according to the instructions of specific kits (Nanjing Jiancheng Bioengineering Institute, China). Serum insulin was measured according to the instructions of Elisa kit (Briggs et al., 2014). The liver tissues were added with lysate and ground with magnetic beads. After centrifugation, the supernatant was taken for the determination of TC and TG according to the instructions.

### Quantification of Protein

Liver and muscle protein lysates (20 mg) were subjected to polyacrylamide gel electrophoresis under reducing conditions followed by transfer to polyvinylidene difluoride membranes. The membranes were incubated with 5% skimmed milk followed by antibodies specific for p-AKT (Cell Signaling Technology, Danvers, MA, United States), AKT (Cell Signaling Technology, Danvers, MA, United States), p-IR (Cell Signaling Technology, Danvers, MA, United States), IR (Cell Signaling Technology, Danvers, MA, United States), and β-ACTIN (Sigma-Aldrich, Shanghai, China). Membranes were then incubated with horseradish peroxidase-conjugated secondary antibodies. The signals were detected using an enhanced chemiluminescence (ECL) system with Pierce SuperSignal West Pico chemiluminescent substrates (Biyuntian Biotechnology, Shanghai, China).

### Histological Evaluation on the Degree of Hepatic Steatosis

Liver tissues were fixed with 10% neutral formalin for 24 h, embedded in paraffin, stained with hematoxylin–eosin staining (H&E), and sections were observed for the degree of hepatic steatosis under the light microscope. The degree of hepatic steatosis was evaluated according to a previous publication in a blinded way (Kleiner et al., 2005). The criteria for scoring include 0 (absent), 1 (rare), 2 (mild), 3 (moderate), and 4 (severe).

### 16S rRNA Sequencing

Fecal DNA was isolated using the Qiagen QIAamp DNA Stool Mini Kit (Qiagen, Dusseldorf, Germany). Illumina sequencing was done based on published methods (Ma et al., 2020). The V3–V4 region of the 16S ribosomal RNA gene was amplified and sequenced. Sequence reads were analyzed using QIIME software 1.9.1 (Caporaso et al., 2010). Functional profiles of microbial communities for 16S rRNA sequencing were predicted using PICRUSt2 (Phylogenetic Investigation of Communities by Reconstruction of Unobserved States). Sequence data associated with this project have been deposited in the NCBI Short Read Archive (SRA) database (Accession Number: PRJNA702866).

### Metagenomics

Total genomic DNA was extracted from fecal samples using the E.Z.N.A. tissue DNA Kit (Omega Bio-tek, Norcross, GA, United States) according to the manufacturer’s instructions. The concentration and purity of extracted DNA were determined with TBS-380 and NanoDrop 2000, respectively. DNA extract quality was checked on 1% agarose gel. Read analysis was performed according to our previous publication (Hong et al., 2020). Adapter sequences were stripped from the 3′ and 5′ end of paired-end Illumina reads using SeqPrep^1^. Reads with length <50 bp or a quality value <20 or having N bases would be considered as low-quality and be removed by Sickle^2^. The hit would be removed if it was associated with the reads, which was aligned to the Rattus norvegicus genome by BWA,^3^ or their mated reads. MEGAHIT, which utilized succinct de Bruijn graphs, was used to assemble data^4^ (Li et al., 2015). Only contigs with the length being or over 300 bp were selected as the final assembling result and were used for further gene prediction and annotation. Open reading frames (ORFs) from each assembled contig were predicted using MetaGene^5^ (Noguchi et al., 2006). The predicted ORFs with length being or over 100 bp were retrieved and translated into amino acid sequences using the NCBI translation table^6^. Then, CD-HIT would be used for clustering predicted genes with a 95% sequence identity (90% coverage^7^) (Fu et al., 2012); the longest sequences from each cluster were selected as representative sequences to construct a nonredundant gene catalog. Reads after quality control were mapped to the representative sequences with 95% identity using SOAPaligner^8^ (Li et al., 2008), and gene abundance in each sample was evaluated. The Kyoto Encyclopedia of Genes and Genomes (KEGG) annotation was conducted using BLASTP (Version 2.2.28+) against the KEGG database (Xie et al., 2011^9^) with an e-value cutoff of 1e^–5^. The higher-order functional information is stored in the KEGG PATHWAY database, which contains three different classification levels (Kanehisa and Goto, 2000). In this study, we focused on KEGG pathway level 1 and level 3. Sequence data have been deposited in the NCBI SRA database (Accession Number: PRJNA719272).

### RNA Sequencing Analysis

Total RNA was extracted from the liver using TRIzol Reagent according to the manufacturer’s instructions (Invitrogen), and genomic DNA was removed using DNase I (TaKara). Then, RNA quality was determined by 2100 Bioanalyzer (Agilent) and quantified using ND-2000 (NanoDrop Technologies). Only the high-quality RNA sample was used to construct the sequencing library. Illumina HiSeq X Ten/NovaSeq 6000 Sequencing and reading were finished based on the published method (Ma et al., 2020). Sequence data were deposited in the NCBI SRA database (Accession Number: PRJNA713932).

### Bioinformatics and Statistical Analysis

Shannon index and Simpson index are the measures of biodiversity (α diversity). Shannon index is intended to quantify both richness and evenness of the species/individuals in the ecosystem or community (Shannon, 1997). Shannon index was calculated as follows: $H_{shannon}=-\sum_{i=1}^{S_{obs}}\frac{n_{i}}{N}ln\frac{n_{i}}{N}$. Based on the assumption on the probability of obtaining similar species samples randomly in an infinite community, Simpson index takes into account the number of species present, as well as the abundance of each species (Simpson, 1949). Simpson index is calculated as follows: $D_{simpson}=\frac{\sum_{i=1}^{S_{obs}}n_{i}\left(n_{i}-1\right)}{N\left(N-1\right)}$, where S_*obs*_ = the number of OTUs actually observed; n_*i*_ = the number of sequences contained in the ith OTU; and N = number of all sequences. Bray–Curtis as the distance algorithm is used to represent β diversity; its calculation is based on the independent taxa (such as OTU, genus, etc.), the weighted calculation method is adopted, and the existence and abundance of species are considered at the same time, but without considering the evolutionary relationship or association information among species (Beals, 1984). The Bray–Curtis dissimilarity index was calculated as follows: $D_{Bray-Curtis}=1-2\frac{\sum \text{min}\left(S_{A,i},S_{B,i}\right)}{\sum S_{A,i}+\sum S_{B,i}}$, where S_*A,i*_ = the number of sequences contained in the ith taxon in sample A; S_*B,i*_ = the number of sequences contained in the ith taxon in sample B. Data shown in this study are expressed as mean ± standard error of mean (SEM) unless otherwise noted. Differences between groups at microbiota phylum and genus levels were calculated by the Mann–Whitney U test. Other comparisons were calculated by two-tailed Student’s *t*-test. *p* < 0.05 was considered statistically significant.

## Results

### Short-Term Antibiotic Exposure Divergently Alters the Composition and Function of Gut Microbiota

**FIGURE 1:**
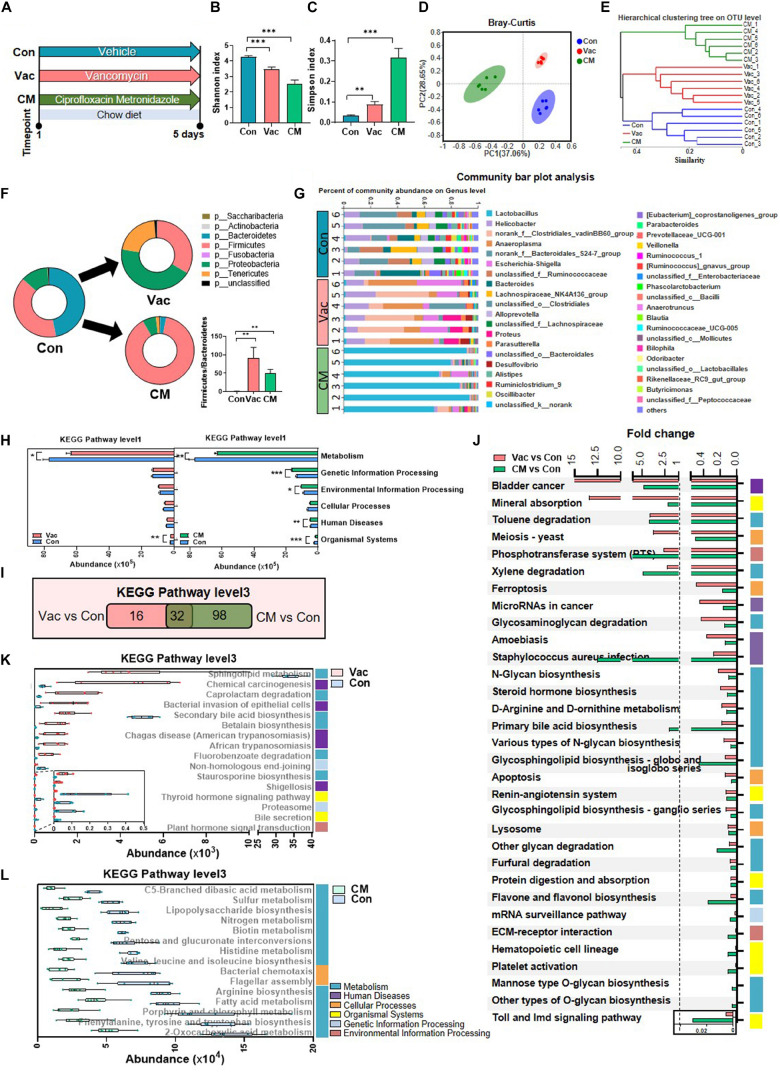
The effects of Vac and CM intervention on regulating gut microbiota composition and function. Rats were given vancomycin (Vac) or combination of ciprofloxacin and metronidazole (CM) by oral gavage twice daily. Rats in the control group (Con) were orally administrated with water. **(A)** Intervention process chart. **(B)** Shannon index. **(C)** Simpson index. **p < 0.01, ***p < 0.001 by Student’s *t*-test. **(D)** Principal co-ordinate analysis based on the Bray–Curtis distance algorithm on OTU level. **(E)** Hierarchical tree of sample clustering. **(F)** Fecal microbiota at phylum level by 16S rRNA sequencing. **(G)** Fecal microbiota at the genus level. **(H)** The function differences between Vac vs. Con and CM vs. Con on KEGG pathway level 1 predicted by PICRUSt2. *p < 0.05, **p < 0.01, and ***p < 0.001 by the Mann–Whitney U test. **(I)** Venn diagram of the overlap of differential enriched pathways between Vac vs. Con and CM vs. Con by PICRUSt2 analysis on KEGG pathway level 3 (*p* < 0.01 under the Mann–Whitney U test, fold change (FC) <0.5 or >2). **(J)** The 32 common pathways between Vac vs. Con and CM vs. Con. **(K)** The 16 unique pathways between Vac and Con groups. **(L)** Top 15 unique pathways between CM and Con groups. *n* = 6 per group.

SD rats were given vancomycin (Vac group), combination of ciprofloxacin and metronidazole (CM group), or water (Con group) twice daily for 5 days on CD (Figure 1A). The 16S rDNA amplicon sequencing of fecal bacteria showed reduced richness and evenness after antibiotic interventions (Figures 1B,C). The three groups clustered separately as shown by Bray–Curtis analysis and hierarchical cluster tree on OTU level, suggesting a significant alternation of the gut microbiota structure (Figures 1D,E). Compared with the Con group, the Vac group had increased relative abundance of Proteobacteria and Tenericutes and reduced Bacteroidetes, while the CM group showed increased relative abundance of Firmicutes and reduced Proteobacteria and Bacteroidetes, resulting in an increased Firmicutes-to-Bacteroidetes ratio in both Vac and CM groups (Figure 1F). At the genus level, the relative abundance of 36 genera were differentially altered by both Vac and CM, while Vac uniquely altered 18 genera (11 upregulated and 7 downregulated) and CM uniquely altered 5 genera (4 upregulated and 1 downregulated) (Supplementary Figure 1). Among them, the dominant bacteria were changed from *Norank_f_Bacteroidates_S24-7_group* (19.4%) and *Helicobacter* (10.3%) in the Con group to *Norank_f_clostridiates_vadinBB60_group* (23.8%) and *Anaeroplasma* (20.5%) in the Vac group (Figure 1G). Most strikingly, the proportion of *Lactobacillus* genus increased to 81% after CM intervention.

These compositional and diversity changes of gut microbiota induced by Vac or CM were accompanied by functional alternations. Based on the KEGG pathway at level 1, Vac intervention impacted metabolism and organismal systems, whereas CM intervention affected all pathways except cellular processes (Figure 1H). At KEGG level 3, CM induced more pathway changes (*p* < 0.01, fold change (FC) < 0.5 or >2) compared with Vac-induced changes (130 vs. 48 pathways) (Figure 1I). Among them, 32 pathways were found to be in common between Vac- and CM-induced pathway changes, and half of them were metabolic pathways (Figure 1J). In addition, most of the 16 unique pathway changes caused by Vac intervention were related to metabolism and human diseases based on KEGG pathway analysis at level 1 (Figure 1K), while the top 15 of CM-induced pathways were metabolic pathways including amino acid biosynthesis and fatty acid metabolism (Figure 1L). In summary, short-term intervention with Vac or CM significantly decreased microbial diversity and altered potential functions of gut microbiota, in which CM had more impact on regulating gut microbial function than Vac in rats.

### Short-Term Antibiotic Exposure Affects Insulin-Signaling Pathway

**FIGURE 2:**
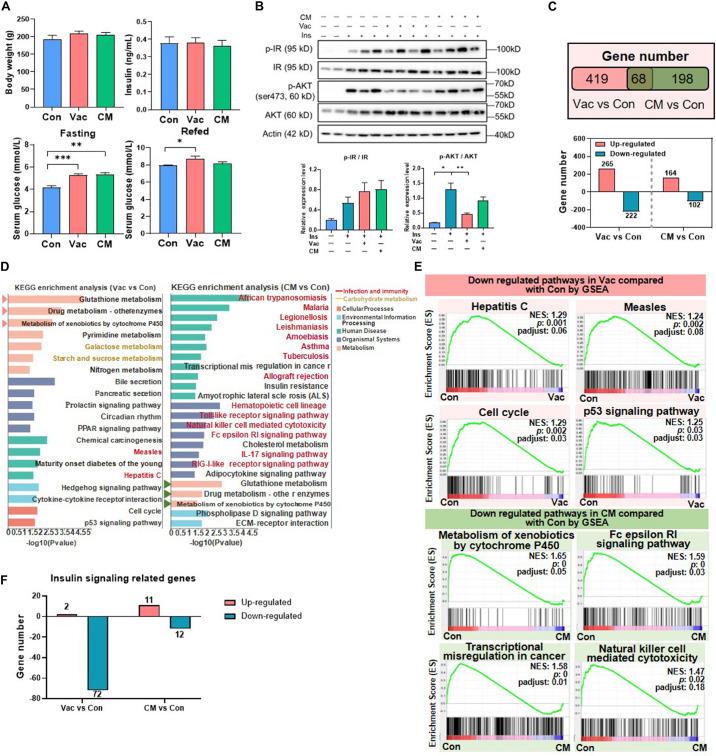
The impact of antibiotic intervention on rat phenotype and liver transcriptome. **(A)** Body weight, fasting serum insulin, fasting serum glucose, and fed serum glucose after antibiotic intervention. **(B)** Western blot analysis of hepatic protein level. Rats were injected with insulin 8 min before tissues were collected. For index determination, *n* = 5–6 per group; for protein analysis, *n* = 2–4 per group; *p < 0.05, **p < 0.01, and ***p < 0.001 by Student’s *t*-test. **(C)** Venn diagram of the overlap of differential expressed genes between Vac vs. Con and CM vs. Con (*p* < 0.05 based on edgeR software, and FC ≤0.5 or ≥2). **(D)** KEGG pathway enrichment analysis of 487 and 266 differential genes. Triangles on the left showed the same pathways between Vac vs. Con and CM vs. Con. **(E)** The same pathways between Gene Set Enrichment analysis (GSEA) and KEGG pathway enrichment analysis. **(F)** Different regulatory effects of Vac and CM on insulin related genes. *n* = 4 per group for transcriptome.

Since the influences of antibiotic intervention on glucose tolerance have been previously reported (Suez et al., 2014; Vrieze et al., 2014; Fujisaka et al., 2016), we expected to test whether the impacts on glucose homeostasis were different when different antibiotics regimens were applied. Results showed that the fasting serum insulin level was comparable among the three groups, whereas the fasting serum glucose levels were higher in both Vac and CM groups than in the Con group (Figure 2A). Interestingly, the serum glucose level of the Vac group remained higher than that of the Con group, but was normalized in CM group 2 h after feeding (Figure 2A). These findings suggested that short-term exposure of Vac and CM affected glucose homeostasis under fasting condition, while Vac caused worse glucose control compared to CM under fed conditions.

Impaired insulin signaling is the important cause for dysglycemia (Kubota et al., 2017). Thus, the expression of proteins involved in insulin signaling pathways was measured in rats that were challenged with insulin injection in the context of either Vac or CM pretreatment. The results showed that insulin increased the protein levels of both phosphate-insulin receptor (p-IR) and IR, resulting in an increased trend of the p-IR/IR ratio in the liver, and this trend was not changed by either Vac or CM. Meanwhile, insulin challenge also significantly elevated the expression of both p-AKT and AKT, as well as the p-AKT/AKT ratio. Vac, not CM, pretreatment markedly reduced p-AKT expression leading to a decreased p-AKT/AKT ratio, suggesting impaired hepatic insulin signaling in Vac-pretreated rats (Figure 2B). In contrast, Vac or CM pretreatment did not affect the insulin signaling pathway in the gastrocnemius (Supplementary Figure 2B). Together, although the fasting insulin levels were comparable among the three groups, Vac and CM elevated the fasting blood glucose level, demonstrating that impaired insulin signaling occurred in Vac- and CM-treated rats. Further re-fed experiment and reduced hepatic p-AKT level and p-AKT/AKT ratio suggested impaired insulin signaling under fed conditions, especially in the Vac group.

### Short-Term Antibiotic Exposure Alters Hepatic Transcriptome

Given the critical roles of gut microbiota in host metabolism (Fan and Pedersen, 2021), we further compared the impacts of short-term Vac or CM pretreatment on host metabolism by using the hepatic transcriptome approach. Compared with the Con group, Vac pretreatment induced 487 differentially expressed genes, while there were only 266 genes that were regulated in the CM group (Figure 2C). The KEGG enrichment analysis of these differentially expressed genes revealed that 20 and 24 pathways were significantly altered by Vac and CM, respectively (Figure 2D). The top three altered metabolic pathways in the Vac group were also found to be changed in CM. Additionally, Vac regulated carbohydrate metabolism, such as galactose metabolism and starch and sucrose metabolism. CM mainly affected infection and immunity-related pathways, as well as insulin resistance and cholesterol metabolism.

To further explore Vac- and CM-induced changes of biological function, Gene Set Enrichment Analysis (GSEA) was employed to identify significantly enriched biological pathways on the basis of normalized enrichment score (NES) ranking. Compared with the Con group, Vac and CM regulated 67 and 44 gene sets (NES > 1 or <-1, *p* < 0.05, *p*-adjust < 0.25), respectively (Supplementary Figures 2C–E). Some pathways that were shifted by both KEGG enrichment analysis and GSEA are shown in Figure 2E, suggesting the importance and significance of these pathways that respond to different antibiotic treatments. We also noticed that Vac intervention has a more extensive disturbance effect on insulin signaling-related genes than CM intervention, and most genes were downregulated (Figure 2F and Supplementary Figure 2F). Because orally administered vancomycin is poorly absorbed in the gut, we speculate that Vac-induced changes of gut microbiota might be correlated with hepatic insulin dysregulation. In sum, Vac and CM had divergent effects on regulating hepatic gene expression where Vac pretreatment affected more gene expression than CM. The metabolic pathways were mainly altered by Vac, whereas pathways of  infection and immunity were predominantly changed by CM.

### Gut Microbiota Composition and Function Are Partly Restored After 45 Days Recovery on Chow Diet

**FIGURE 3:**
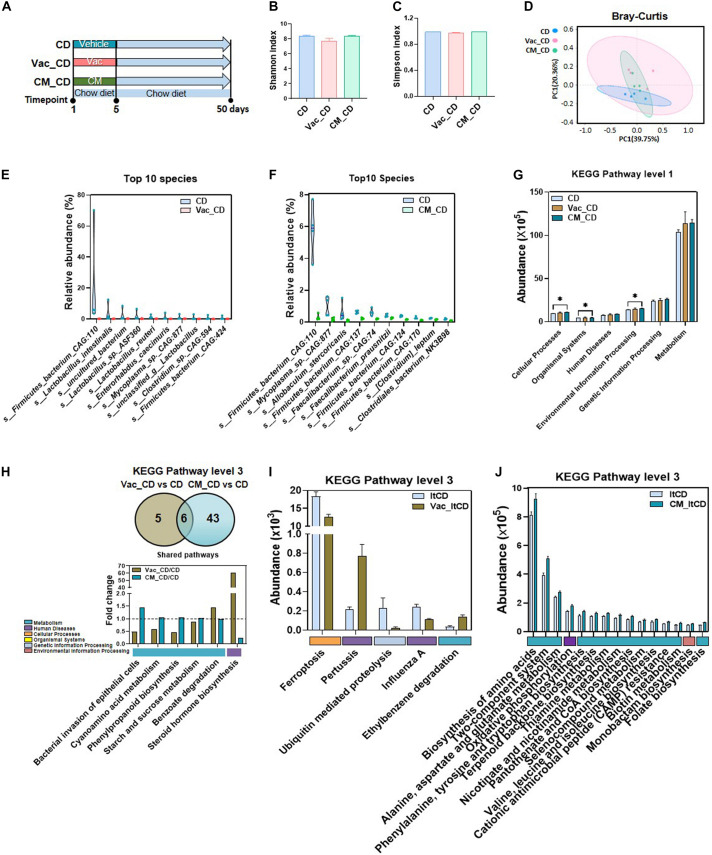
The long-lasting impact of antibiotic treatment on bacterial composition and function when rats were fed with chow diet. **(A)** Intervention process chart. Rats were treated with vehicle, Vac, or CM for 5 days and then fed with chow diet for another 45 days. **(B)** Shannon index. **(C)** Simpson index. **(D)** Principal co-ordinate analysis based on the Bray–Curtis distance algorithm on species level. **(E,F)** Top 10 differential species between Vac_CD and CD and between CM_CD and CD (*p* < 0.05 by the Mann–Whitney U test). **(G)** The function differences among groups on KEGG pathway level 1 predicted by PICRUSt2. *p < 0.05 by Student’s *t*-test. **(H)** Venn diagram of the overlap of differential enriched pathways between Vac_CD vs. CD and CM_CD vs. CD by PICRUSt2 analysis on KEGG pathway level 3 (*p* < 0.05 under the Mann–Whitney U test). The overlapped pathways between Vac_CD vs. CD and CM_CD vs. CD. **(I)** The unique shifted pathways between Vac_CD and CD. **(J)** Top 15 unique shifted pathways between CM_CD and CD.

Gut dysbiosis caused by antibiotics can persist for extended periods. To study the recovery extent of gut microbial profile and function after a short-term Vac or CM pretreatment, metagenomic analysis was performed when rats recovered on CD for 45 days (Figure 3A). Although long-term (45 days) CD intake recovered the general structure of gut microbiota in the Vac_CD (post-Vac recovery under CD) and CM_CD (post-CM recovery under CD) groups, revealed by alpha and beta diversity analysis (Figures 3B-D), many bacterial species and potential microbial functions were still significantly different among groups. At the species level, the relative abundance of 63 species and 15 species was different between CD and Vac_CD and between CD and CM_CD (*p* < 0.05), respectively. In particular, four species of top 10 species that changed in the Vac_CD group belonged to *Lactobacillus*, indicating that the difference of *Lactobacillus* might account for the difference of the gut microbiota profile between the Vac_CD and CD groups after recovery (Figure 3E). Additionally, among the significantly changed species in the CM_CD group, four belonged to *Firmicutes bacterium* in CM_CD and two species belonged to *Faecalibacterium*. Moreover, the biggest reduction at species level after recovery was *Firmicutes bacterium* CAG:110, which was consistently reduced in the Vac_CD and CM_CD groups (Figure 3F).

We next predicated the potential function of gut microbiota to study whether bacterial function was restored after recovery from antibiotic exposure. At KEGG level 1, environmental information processing, organismal systems, and cellular processes were increased in the CM_CD group, while no pathway was altered in the Vac_CD group (Figure 3G). At KEGG level 3, 11 and 49 pathways were significantly different between Vac_CD and CD groups and between CM_CD and CD groups (*p* < 0.05), respectively (Figures 3H–J). Pathways of starch and sucrose metabolism and cyanoamino acid metabolism, which were not different after antibiotic perturbation, were found to be increased in both the Vac_CD and CM_CD groups. In addition, the number of differential pathways narrowed after recovery under CD when compared with the number after antibiotic intervention, suggesting partly recovery of intestinal bacterial structure and function in rats.

### Physiological Status After 45 Days Recovery From Antibiotic Exposure on Chow Diet

**FIGURE 4:**
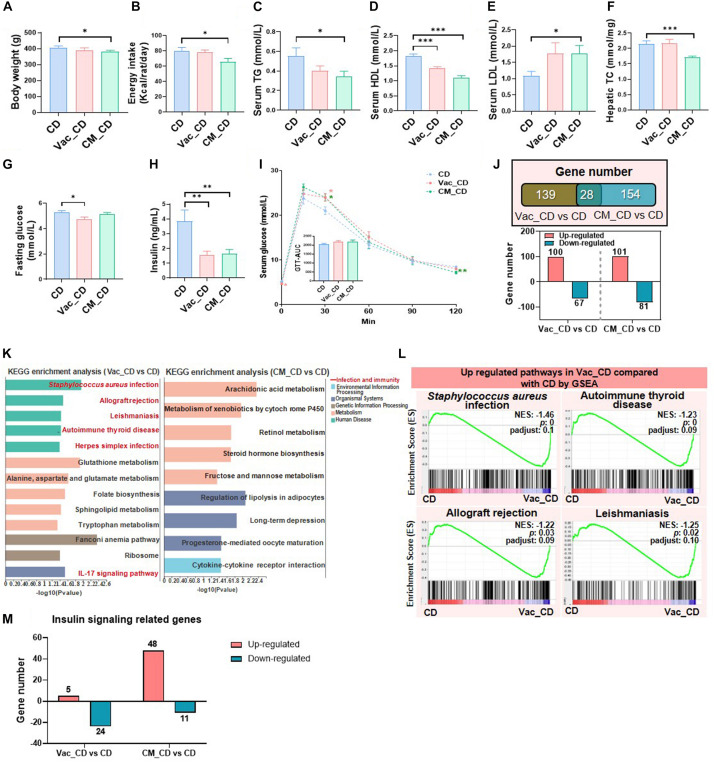
The long-lasting impact of antibiotic treatment on rat phenotype and liver transcriptome when rats were fed with chow diet. The body weight **(A)**, energy intake **(B)**, serum triglyceride level **(C)**, serum high-density lipoprotein level **(D)**, serum low-density lipoprotein level **(E)**, and liver total cholesterol level **(F)** were shown at the end of the 45-day recovery. **(G–I)** Fasting blood glucose level, fasting blood insulin level, and glucose tolerance test result. **(J)** Venn diagram of the overlap of differential expressed genes between Vac_CD vs. CD and CM_CD vs. CD (*p* < 0.05 based on edgeR software, and FC ≤0.5 or ≥2). **(K)** KEGG pathway enrichment analysis of 167 and 182 differential genes. **(L)** The same pathways between Gene Set Enrichment analysis (GSEA) and KEGG pathway enrichment analysis. **(M)** Different regulatory effects of Vac and CM on insulin-related genes. *n* = 9–10 per group for biochemical analysis, *p < 0.05, **p < 0.01, and ***p < 0.001 by Student’s *t*-test; *n* = 3 per group for transcriptome, Mann–Whitney U test.

Pretreatment with CM reduced energy intake during the prolonged CD feeding, resulting in slightly lower BW compared to the CD group (Figures 4A,B). In addition, the levels of serum TG and HDL tended to be decreased in the Vac_CD and CM_CD groups, while serum LDL tended to be increased (Figures 4C–E). Moreover, CM_CD has reduced the hepatic TC level, while the levels of serum TC, LPS, hepatic TG, and liver histology were comparable between groups (Figure 4F and Supplementary Figures 3A,B). The fasting glucose level was a little bit lower in the Vac_CD group, and the fasting insulin levels were significantly lower in both Vac_CD and CM_CD groups (Figures 4G,H). However, the area under the curve (AUC) of the glucose tolerance test revealed a similar glucose clearance capacity in the three groups (Figure 4I). These findings suggested the enhanced insulin capacity of regulating glucose homeostasis in the Vac_CD and CM_CD groups. Post-antibiotic recovery of gut microbiota reversed impaired insulin signaling in rats fed with CD.

### Liver Gene Expression Is Partly Restored After 45 Days Recovery on Chow Diet

Since gut microbiota composition was partly restored after 45 days CD feeding, we next assessed the status of host metabolism by analyzing the hepatic transcriptome. First of all, the number of differentially expressed genes was comparable between Vac_CD and CM_CD groups, both of which were greatly reduced after 45 days recovery on CD (Figures 2C, 4J). The KEGG enrichment analysis revealed that 13 pathways were altered in the Vac_CD group (*p* < 0.05) (Figure 4K). However, nine pathways were enriched in the CM_CD group (*p* < 0.05), four of which were related to sugar and lipid metabolism, such as arachidonic acid metabolism, steroid hormone biosynthesis, fructose and mannose metabolism, and regulation of lipolysis in adipocytes (Figure 4K). Meanwhile, four pathways were commonly found by KEGG enrichment analysis and GSEA in the Vac_CD group, including *Staphylococcus aureus* infection, allograft rejection, leishmaniasis, and autoimmune thyroid disease (Figure 4L and Supplementary Figure 3C). Interestingly, post-Vac recovery on CD increased the number of differentially expressed genes of the insulin-related pathway when compared with short-term Vac intervention, while post-CM recovery on CD had the opposite effect (Figures 2F, 4M). In addition, the number and specific expression panel of altered genes in this pathway were largely different in the Vac_CD or CM_CD group, where more genes were kept upregulated in CM_CD but downregulated in the Vac_CD group compared with the CD group (Figure 4M and Supplementary Figure 3D). Altogether, these results suggested that the host metabolic responses to short-term antibiotic exposure were long-lasting and antibiotic-specific.

### The Recovery of Gut Dysbiosis After the 45-Day High-Fat Diet Feeding

**FIGURE 5:**
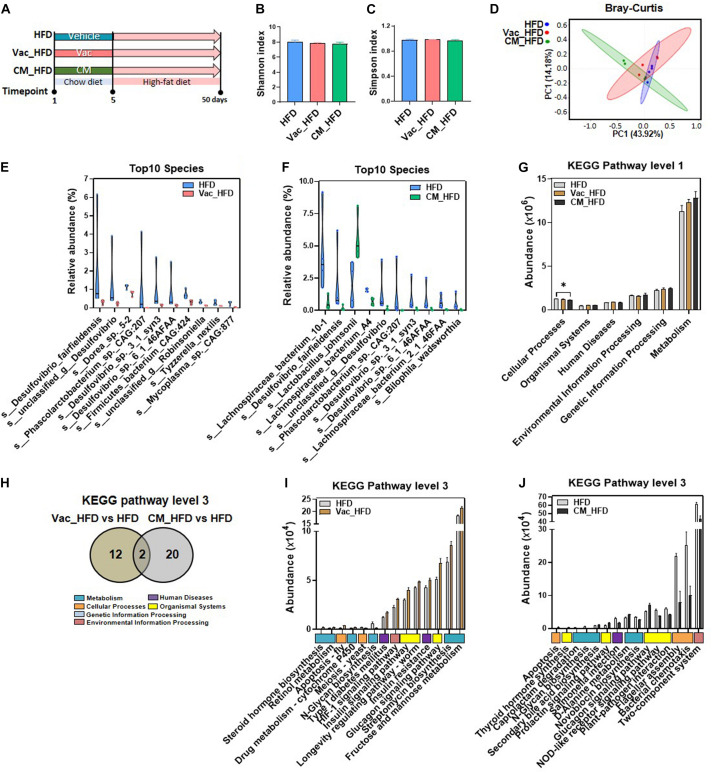
HFD affects post-antibiotic recovery of gut microbiota composition and function. **(A)** Intervention process chart. Rats were treated with vehicle, Vac, or CM for 5 days and then fed with a high-fat diet for another 45 days. **(B)** Shannon index. **(C)** Simpson index. **(D)** Principal co-ordinate analysis based on the Bray–Curtis distance algorithm at species level. **(E,F)** Top 10 differential species between Vac_HFD and HFD and between CM_HFD and HFD (*p* < 0.05 by the Mann–Whitney U test). **(G)** The function differences among groups on KEGG pathway level 1 predicted by PICRUSt2. *p < 0.05 by Student’s *t*-test. **(H)** Venn diagram of the overlap of differential enriched pathways between Vac_HFD vs. HFD and CM_HFD vs. HFD by PICRUSt2 analysis on KEGG pathway level 3 (*p* < 0.05 under the Mann–Whitney U test). **(I)** The shifted pathways between Vac_HFD and HFD. **(J)** Top 15 shifted pathways between CM_HFD and HFD.

High-fat diet is an important factor for influencing gut microbiota composition resulting in the changes of host metabolism (Gentile and Weir, 2018; Zmora et al., 2019). To further evaluate the dietary impact on post-antibiotic recovery, rats were switched to HFD for 45 days after a short-term antibiotic intervention (Figure 5A). In general, similar with the results after recovery on CD, the Shannon and Simpson indices as well as PCoA results showed no significant difference among HFD, Vac_HFD (post-Vac recovery under HFD), and CM_HFD (post-CM recovery under HFD) groups (Figures 5B–D). At species level, the number of significant differential species between pre-Vac-treated and untreated groups was dramatically reduced from 63 when rats were recovered on CD to 14 when recovered on HFD, while the differential number between pre-CM-treated and untreated groups was increased from 15 when recovered on CD to 21 when recovered on HFD. Among the top 10 significant differential species between Vac_HFD and HFD groups, four species belonged to *Desulfovibrio*, indicating the importance of *Desulfovibrio* for the difference of gut microbiota structure during recovery on HFD (Figure 5E). In addition, the CM_HFD group had markedly increased *s_Lactobacillus_johnsonii* (the fold change was 2.85) and decreased three species of *Lachnospiraceae_bacterium* and three species of *Desulfovibrio* compared to the HFD group (Figure 5F).

Further prediction on the potential function of gut microbiota demonstrated that the cellular process pathway was reduced in the CM_HFD group compared to the HFD group at KEGG pathway level 1 (Figure 5G). At level 3, the number of altered pathways was greatly reduced after recovery on HFD than recovery on CD. Vac_HFD and CM_HFD only altered 14 and 22 pathways, respectively, in which two pathways were commonly changed by both (Figure 5H). The most significant changes after recovery on HFD was the induction of sugar metabolism and insulin signaling-related pathways in Vac_HFD, including fructose and mannose metabolism, glucagon signaling pathway, insulin resistance, and insulin signaling pathway, while the glucagon signaling pathway was increased in both Vac_HFD and CM_HFD (Figures 5I,J). Different with the findings that most KEGG pathways were elevated in Vac_HFD, most pathways were decreased in CM_HFD compared to the HFD group. Together, the results suggested that the gut dysbiosis which resulted from short-term Vac or CM intervention was long-lasting and antibiotic-related after the 45-day recovery on HFD feeding.

### The Recovery of Glucose Homeostasis After the 45-Day High-Fat Diet Feeding

**FIGURE 6:**
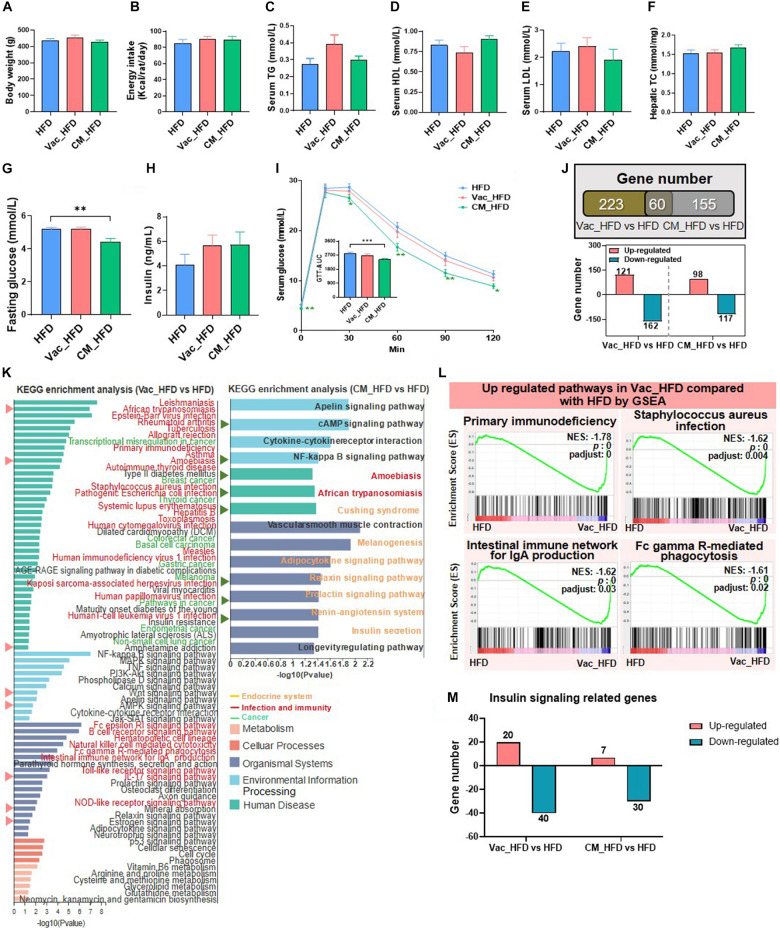
High-fat diet affects post-antibiotic recovery of rat phenotype and hepatic transcriptome. The body weight **(A)**, energy intake **(B)**, serum triglyceride level **(C)**, serum high-density lipoprotein level **(D)**, serum low-density lipoprotein level **(E)**, and liver total cholesterol level **(F)** were shown at the end of the 45-day recovery. **(G–I)** Fasting blood glucose level, fasting blood insulin level, and glucose tolerance test result. **(J)** Venn diagram of the overlap of differential expressed genes between Vac_HFD vs. HFD and CM_HFD vs. HFD (*p* < 0.05 based on edgeR software, and FC ≤0.5 or ≥2). **(K)** KEGG pathway enrichment analysis of 283 and 215 differential genes. Triangles on the left showed the same pathways between Vac_HFD vs. HFD and CM_HFD vs. HFD. **(L)** The same pathways between Gene Set Enrichment analysis (GSEA) and KEGG pathway enrichment analysis. **(M)** Different regulatory effects of Vac and CM on insulin-related genes. *n* = 9–10 per group for biochemical analysis, **p < 0.01 and ***p < 0.001 by Student’s *t*-test; *n* = 3 per group for transcriptome, Mann–Whitney U test.

Since recovery on HFD shifted sugar metabolism and insulin signaling-related pathways, we further studied the phenotype changes after long-term HFD feeding post-antibiotic intervention. There was no difference among the three groups regarding BW and energy intake post-antibiotic recovery on HFD (Figures 6A,B). Different from the obvious changes of lipid profiles in serum and liver observed during post-antibiotic recovery on CD, HFD feeding after antibiotic exposure had little effects on regulating these phenotypes (Figures 6C–F and Supplementary Figures 4A,B). Although short-term Vac intervention reduced fasting blood glucose when rats were recovered on CD, it had little effect on improving glucose homeostasis when recovered on HFD. By contrast, short-term CM intervention followed by HFD feeding lowered the fasting blood glucose level and improved glucose tolerance as revealed by the decreased AUC in CM_HFD (Figures 6G,I). In addition, fasting insulin levels were slightly higher in Vac_HFD and CM_HFD groups without significant difference (Figure 6H). These findings suggested that different early antibiotic exposures divergently affected glycemia at the recovery stage on HFD.

### The Recovery of Hepatic Gene Expression After Antibiotic Exposure Is Affected by High-Fat Diet

The number of differential expressed genes between groups was markedly increased when rats were recovered on HFD than on CD. Specifically, the expressions of 283 and 215 hepatic genes were changed by Vac_HFD and CM_HFD, respectively, in which more genes were downregulated (Figure 6J). KEGG enrichment analysis revealed that 78 pathways were shifted between Vac_HFD and HFD (*p* < 0.05), while there were only 13 differential pathways when comparing Vac_CD with CD, suggesting that HFD feeding enlarged short-term Vac treatment-induced changes of liver function at the recovery condition. Among them, 30 pathways were related to infection and immunity and 10 pathways were associated with cancer (Figure 6K). However, only 15 pathways were shifted between CM_HFD and HFD (*p* < 0.05) and 7 of them were associated with the endocrine system (Figure 6K). GSEA results further showed that 37 gene sets were changed by Vac_HFD, many of which were related to infectious disease or the immune system (Figure 6L and Supplementary Figures 4C–E). Regarding insulin signaling-related genes, when compared with the number of differential genes between CM_CD and CD (most genes were upregulated in the CM_CD group), HFD intake reduced the number of differential genes between CM_HFD and HFD, with most genes downregulated in both cases (Figure 6M and Supplementary Figure 4F). These results might correlate with the improved glucose tolerance in the CM_HFD group. These results might correlate with the improved glucose tolerance in the CM_HFD group. Together, short-term Vac and CM interventions have unique long-term effects on modulating liver function. The long-lasting effects of early Vac and CM perturbation after recovery on HFD were associated with the changes of infection, immunity, cancer-related pathways, and endocrine system-related pathways, respectively.

### Post-antibiotic Recovery on HFD Enlarges Antibiotic-Induced Metabolic Differences

**FIGURE 7:**
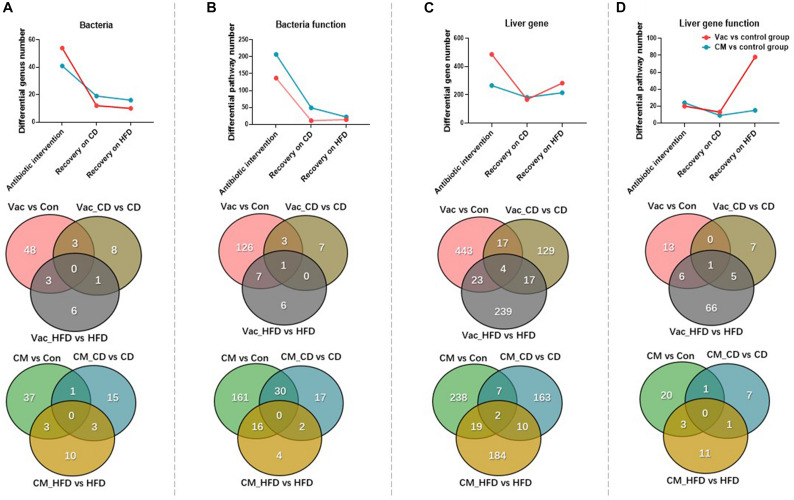
The changes of gut microbiota and liver gene after antibiotic intervention and post-antibiotic recovery. **(A–D)** Venn diagram and line chart show the number changes of differential genus, functional prediction of bacteria at KEGG pathway level 3, differential expression of liver genes, and functional prediction of genes among the three conditions under the same screening conditions.

To investigate how diet affects host recovery after different antibiotic perturbations, we compared the composition and function of gut microbiota as well as the gene expression and biological function of the liver after short-term Vac and CM exposure and at post-antibiotic recovery state when rats were fed with CD or HFD. After short-term antibiotic intervention, compared with the control group, the number of differential genera was higher in Vac than in the CM group. However, the differential genus number was dramatically reduced after recovery on both CD and HFD with a slightly higher number in CM than in Vac (Figure 7A). The Venn diagram showed that only a few genera were commonly altered by any two conditions with no genus commonly regulated by three conditions. Although short-term Vac induced more changes at the genus level, CM affected more pathway changes of the bacterial function pathway, and the differential pathway number was reduced post-antibiotic recovery, suggesting partly recovery of gut bacterial function (Figure 7B and Supplementary Figure 5).

Liver gene expression was affected more significantly by Vac than by CM based on the number of regulated genes (Figure 7C). Post-antibiotic recovery with CD reduced the number of differential expression genes, but recovery with HFD increased this number especially in the Vac_HFD group (Figure 7C). In addition, the shifted pathway number between Vac- and CM-induced changes was comparable in short-term antibiotic intervention and recovery on CD condition. However, post-Vac recovery on HFD markedly elevated the differential pathway number to 78, in which most pathways were infection-, immunity-, and cancer-related, whereas only 15 pathways were changed with post-CM recovery on HFD, and most of them were endocrine system-related (Figure 7D). Glutathione metabolism was the only pathway affected by all three conditions with Vac perturbation (Supplementary Tables 1, 2). These results suggested that although gut microbiota composition and function were partly restored from antibiotic exposure, recovery on HFD, but not on CD, significantly increased the impact of early Vac exposure on host metabolism.

## Discussion

**FIGURE 8:**
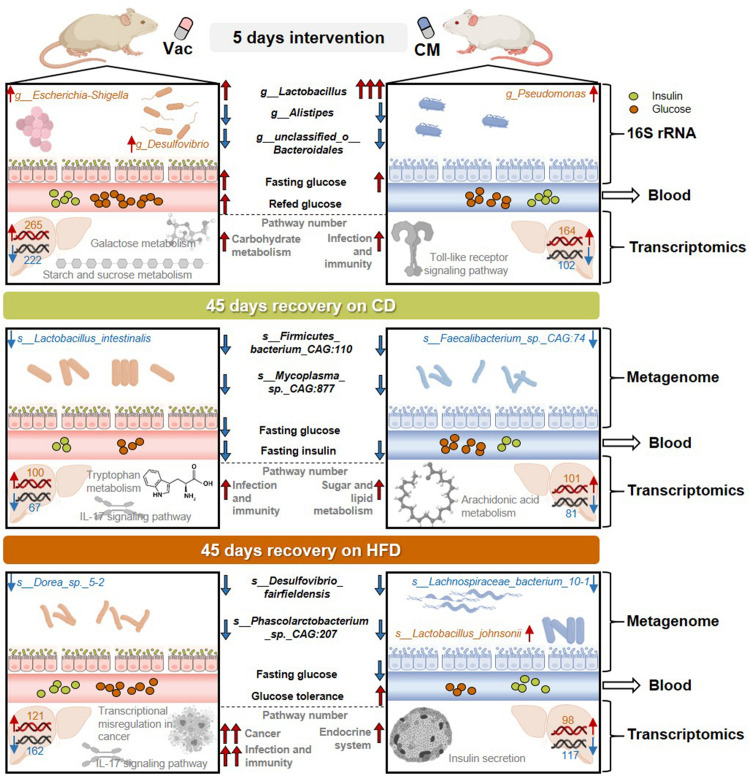
The effects of short-term antibiotic intervention on gut microbiota composition and host metabolism were long-lasting, antibiotic-specific, and diet-dependent. The 5-day intervention with Vac and CM dramatically influenced gut microbiota composition, in which Vac and CM had unique or common effects on regulating the relative abundance of certain gut bacteria. In addition, short-term Vac and CM intervention differential affected blood glucose homeostasis and liver transcriptome, by mainly regulating carbohydrate metabolism-related pathways and infection- and immunity-related pathways, respectively. After a 45-day recovery on either CD or HFD, gut microbiota composition and function, as well as blood glucose imbalance, were partly restored. However, when rats were recovered on CD, certain species in *Lactobacillus* genus and in *Faecalibacterium* genus were reduced at post-Vac and post-CM recovery states, respectively. Differences in infection and immunity-related pathways were apparent after recovery from Vac treatment, while sugar and lipid metabolism-associated pathways were shifted after recovery from CM treatment as revealed by hepatic transcriptome. When rats were recovered on HFD, the relative abundance of species in *Desulfovibrio* and *Lachnospiraceae* genera was reduced, whereas *Lactobacillus johnsonii* was elevated significantly at post-CM recovery state. Moreover, HFD feeding worsened the extent of post-antibiotic recovery in Vac-treated rats including the alterations of infection, immunity, and cancer-related pathways and in CM-treated rats including endocrine system-associated pathways. CD, chow diet; CM, ciprofloxacin and metronidazole; HFD, high-fat diet; Vac, vancomycin.

Gut microbiota composition and diversity exert profound effects on host physiology and metabolism. Mounting evidences have shown that exposures to antibiotics in both animals and humans have transient and prolonged effects on host metabolism homeostasis. As summarized in Figure 8, the current study systematically investigated gut microbiota composition and host metabolism after short-term antibiotic intervention and at post-antibiotic recovery state and found that the impacts of antibiotic exposure on host metabolism were long-lasting, antibiotic-specific, and diet-dependent. These findings suggest that dietary management during post-antibiotic recovery should particularly be given more attention.

Exposure to antibiotics is closely linked with the changes of glucose metabolism in both human and animal studies. A randomized double-blinded study showed that 7-day Vac treatment reduced peripheral insulin sensitivity in obese males with metabolic syndrome (Vrieze et al., 2014), whereas another study found that it did not affect tissue-specific insulin sensitivity in obese, prediabetic males (Reijnders et al., 2016). In an animal study, short-term or long-term Vac intervention reduced fasting blood glucose in obese mice (Fujisaka et al., 2016; Aron-Wisnewsky et al., 2020). It has been reported that not only Vac but also CM combination can improve noncaloric artificial sweetener-induced glucose intolerance in mice (Suez et al., 2014). However, in contrast with several previous studies in rodents, our results showed that 5-day exposure of Vac and CM increased the fasting blood glucose level and Vac alone elevated blood glucose under a re-fed state. In particular, short-term Vac decreased the insulin-induced p-AKT/AKT ratio, suggesting impaired hepatic insulin signaling after 5-day Vac intervention. Inconsistent with our findings, Fujisaka et al. (2016) found that Vac could increase the p-AKT/AKT ratio in the liver and muscle in HFD-fed mice without insulin supplementation, while metronidazole increased p-AKT level in the liver and adipose tissue only in response to insulin. These inconsistent results suggest that the interaction between gut microbiome and insulin signaling is very complex and many factors, such as animal model, diet, and host health conditions, will affect the outcomes. Additionally, changes of bacterial metabolites after antibiotic intervention, such as secondary bile acids and short-chain fatty acids, might play an important role in causing hepatic insulin dysregulation through the gut–liver axis, which warrants further investigation.

Since antibiotic treatment dramatically impacted glucose homeostasis and insulin signaling, to our surprise, little is known about how glucose levels change at post-antibiotic recovery state and whether diet will affect the recovery. Fu et al. (2018) have reported that treatment with antibiotic cocktail for 12 days reduced blood glucose in db/db mice, and this difference disappeared 24 days after antibiotic withdrawal. This result suggested that post-antibiotic recovery reversed antibiotic-induced changes of glucose homeostasis. However, another clinical trial showed that host metabolism, such as whole-body insulin sensitivity, remained unchanged by 7-day Vac perturbation as well as 8 weeks post-intervention (Reijnders et al., 2016). Our data revealed impaired insulin signaling after short-term Vac treatment, while recovery on CD could decrease fasting blood glucose level and insulin level, suggesting enhanced insulin activity at post-Vac recovery state on CD. More importantly, our further investigation found that HFD feeding during recovery increased the fasting insulin level in post-antibiotic groups (1.7 ng/ml in the Vac_CD group and 2 ng/ml in the CM_CD group, 5.6 ng/ml in the Vac_HFD group, and 5.7 ng/ml in the CM_HFD group) but not in the vehicle group (3.8 ng/ml in CD and 4.1 ng/ml in the HFD group), suggesting that early antibiotic exposure enhanced rat susceptibility to HFD-induced changes of fasting insulin level.

Short-term antibiotic perturbations have a long-term effect on gut microbial composition and function. Based on the KEGG pathway analysis, compared with the effect of CD on post-Vac recovery, HFD intake during post-Vac recovery led to functional changes in bacteria which related to glucose homeostasis. This result was in line with the phenotype changes that HFD reversed the CD-induced decrease in fasting insulin level during post-antibiotic recovery. The most significant change of gut microbiota composition induced by 5-day CM treatment was the 127-fold induction of the abundance of the *Lactobacillus* genus. Interestingly, when rats recovered under CD, the abundance of three species from *Lactobacillus* genus, *Lactobacillus intestinalis*, *Lactobacillus reuteri*, and *Lactobacillus* sp. *ASF360*, was reduced in the Vac_CD group, while there was no difference in the *Lactobacillus* species between the CM_CD and CD groups. Previous study reported that *L. intestinalis* was increased in Zucker diabetic fatty rats and can be a potential biomarker for the progression and complications of T2DM (Wang et al., 2020). However, some strains of *L. reuteri* are used as probiotics to improve insulin sensitivity (Mobini et al., 2017; Kolodziej and Szajewska, 2019). These findings suggested that *Lactobacillus* might play an important role in post-Vac recovery of glucose homeostasis. In contrast to the findings in the Vac_CD group, when rats were fed with HFD, the most significant long-term effect of Vac intervention was the reduction of the *Desulfovibrio* genus, which can be boosted by HFD and can produce hydrogen sulfide leading to acute inflammation (Attene-Ramos et al., 2006; Kushkevych et al., 2019; Rohr et al., 2020). Among the top 10 changed bacteria, four species in the *Desulfovibrio* genus were reduced in the Vac_HFD group compared with the HFD group. *Desulfovibrio* has been reported to positively correlate with the AUC of OGTT, insulin, and HOMA-IR (Zhou et al., 2018). Therefore, short-term Vac exposure might affect post-Vac recovery of host glucose homeostasis by regulating *Lactobacillus* abundance and *Desulfovibrio* abundance during long-term CD feeding and HFD feeding, respectively.

Early CM intervention also affected gut microbiota recovery based on diet. Compared with their respective control groups, among the top 10 changes species, two species that belong to the *Faecalibacterium* genus were reduced in the CM_CD group, while three species that belong to the unclassified Lachnospiraceae genus were reduced in the CM_HFD group. *Faecalibacterium prausnitzii*, which is a butyrate-producing bacteria, has anti-inflammatory properties and plays a crucial role in maintaining host physiology (Lopez-Siles et al., 2017). Certain strains of Lachnospiraceae, which also produce butyrate, can regulate host metabolism, immune response, and colonocyte growth (Meehan and Beiko, 2014). Additionally, higher levels of Lachnospiraceae have been negatively associated with the risk of some types of cancer (Flemer et al., 2018). These findings suggested that the long-term effect of CM exposure might be correlated with decreased beneficial butyrate production. Surprisingly, the abundance of *L. johnsonii* was the only significantly increased species in the CM_HFD group than in the HFD group. Because many studies have shown that *L. johnsonii* modulates host immune responses (Fonseca et al., 2017; Marcial et al., 2017; Xia et al., 2020), the elevated *L. johnsonii* might lead to better immune response in the CM_HFD group. In sum, different short-term antibiotic exposures have divergent effects on regulating host physiology and metabolism, which depend on the diet used during the recovery state.

Although few studies have reported altered expressions of hepatic genes and shifted functional pathways long after antibiotic exposure (Cho et al., 2012; Nobel et al., 2015), to our knowledge, little is known about the changes of liver biological functions after short-term antibiotic treatment and post-antibiotic recovery, especially with different diet feedings. Our data showed that short-term Vac exposure, but not CM exposure, induced more profound effects in later life on HFD than on CD, including altered infection, immunity, and cancer-related pathways. Plenty of evidences have shown that HFD-driven dysbiosis was accompanied by a vast expansion of pathogen infection (Zeng et al., 2018; Las Heras et al., 2019; Mefferd et al., 2020). It has been shown that 2 weeks after Vac treatment, mice were still susceptible to pathogen colonization (Isaac et al., 2017). Therefore, our results of hepatic transcriptomics demonstrated that HFD-induced long-lasting effects of Vac treatment on liver function, including altered infection and immunity pathways as well as cancer-related pathways, might be partly due to enhanced susceptibility to pathogen intestinal colonization under HFD feeding.

In conclusion, long-lasting effects of antibiotic exposure on host metabolism are antibiotic-specific and diet-dependent. Our current study reveals the interplay between antibiotic-driven dysbiosis and biological functions of liver, and more importantly, we demonstrate that HFD intake during recovery could worsen Vac-induced long-term detrimental consequences. Although the role of dietary fiber in post-antibiotic recovery of gut microbiome is reported previously (Ng et al., 2019; Tanes et al., 2021), our study evaluates how high-fat diet affects post-antibiotic recovery of host metabolism, which will add our knowledge of the dietary effect on host health. Our finding highlights the importance of dietary management after antibiotic exposure. Further investigations are warranted to find out better dietary types that can reduce antibiotic-induced detrimental consequences, which might be of importance to benefit clinical use for post-antibiotic recovery.

## Data Availability Statement

The original contributions presented in the study are publicly available. This data can be found here: https://www.ncbi.nlm.nih.gov/sra, PRJNA719272, PRJNA713932, and PRJNA702866.

## Ethics Statement

The animal study was reviewed and approved by Shanghai University of Traditional Chinese Medicine Animal Ethics Committee.

## Author Contributions

BL conducted the *in vivo* experiments, data analysis, and manuscript writing. HQ, YH, and JM helped in H&E staining of tissues and hepatic steatosis evaluation. NZ, GW, YG, and JZ helped in the animal experiment. WZ helped in the project design. LS and HL co-supervised the study and wrote the manuscript. HL conceptualized the project. All authors contributed to the article and approved the submitted version.

## Conflict of Interest

The authors declare that the research was conducted in the absence of any commercial or financial relationships that could be construed as a potential conflict of interest.

## Footnotes

1 https://github.com/jstjohn/SeqPrep

2 https://github.com/najoshi/sickle

3 http://bio-bwa.sourceforge.net

4 https://github.com/voutcn/megahit

5 http://metagene.cb.k.u-tokyo.ac.jp/

6 http://www.ncbi.nlm.nih.gov/Taxonomy/taxonomyhome.html/index.cgi?chapter=tgencodes#SG1

7 http://www.bioinformatics.org/cd-hit/

8 http://soap.genomics.org.cn/

9 http://www.genome.jp/keeg/

## Supplementary Material

Supplementary Figure 1 Differential bacteria between antibiotic intervention group and control group at genus level (*p* < 0.01 under Mann–Whitney U test).

Supplementary Figure 2 The impact of antibiotic intervention on rat phenotype and liver transcriptome. **(A)** Serum ALT, AST, LDL, and HDL levels after Vac and CM innervation. **(B)** Western blot analysis of protein level in gastrocnemius. Rats were injected with insulin 8 min before tissues collected. **(C)** Venn diagram of the overlap of the differential enriched gene sets between Vac vs. Con and CM vs. Con based on GSEA (NES > 1 or <-1, *p* < 0.05, *p*-adjust < 0.25). **(D)** The common gene sets that were regulated by both Vac and CM based on GSEA result. **(E)** The gene sets that were regulated by either Vac or CM based on GSEA result. **(F)** Heatmaps show the different regulatory effects of Vac and CM on insulin related genes. *n* = 4 per group for transcriptome.

Supplementary Figure 3 The long-lasting impact of antibiotics treatment on rat phenotype and liver transcriptome when rats were fed with chow diet. **(A)** Serum LPS, serum TC, hepatic TG level. **(B)** H&E-stained liver sections and HE score. **(C)** Venn diagram of the overlap of the differential enriched gene sets between Vac_CD vs. CD and CM_CD vs. CD based on GSEA (NES > 1 or <-1, *p* < 0.05, *p*-adjust < 0.25). The gene sets that were regulated by either Vac or CM based on GSEA result. **(D)** Heatmaps show the different regulatory effects of Vac and CM on insulin related genes. *n* = 3 per group for transcriptome.

Supplementary Figure 4 HFD affects post-antibiotic recovery of rat phenotype and hepatic transcriptome. **(A)** Serum LPS, serum TC, hepatic TG level. **(B)** H&E-stained liver sections and HE score. **(C)** Venn diagram of the overlap of the differential enriched gene sets between Vac_HFD vs. HFD and CM_HFD vs. HFD based on GSEA (NES > 1 or <-1, *p* < 0.05, *p*-adjust < 0.25). **(D)** The common gene sets that were regulated by both Vac_HFD and CD_HFD. **(E)** The gene sets that were regulated by either Vac_HFD or CM_HFD based on GSEA result. **(F)** Heatmaps show the different regulatory effects of Vac and CM on insulin related genes. *n* = 3 per group for transcriptome.

Supplementary Figure 5 Shared bacterial functional pathways among three conditions. **(A)** The 11 shared pathways between Vac vs. Con, Vac_CD vs. CD and Vac_HFD vs. HFD on KEGG pathway level 3 were shown with heatmap (Log_2_ Fold change). **(B)** The 48 shared pathways between CM vs. Con, CM_CD vs. CD and CM_HFD vs. HFD on KEGG pathway level 3 were shown with heatmap (Log_2_ Fold change).
