# Supplementary figures and images for: Integrated Metagenomic and Transcriptomic Analyses Reveal the Dietary Dependent Recovery of Host Metabolism From Antibiotic Exposure
Source: Front Cell Dev Biol. 2021 Jun 18;9:680174. doi: 10.3389/fcell.2021.680174 (PMC8250461; doi:10.3389/fcell.2021.680174)

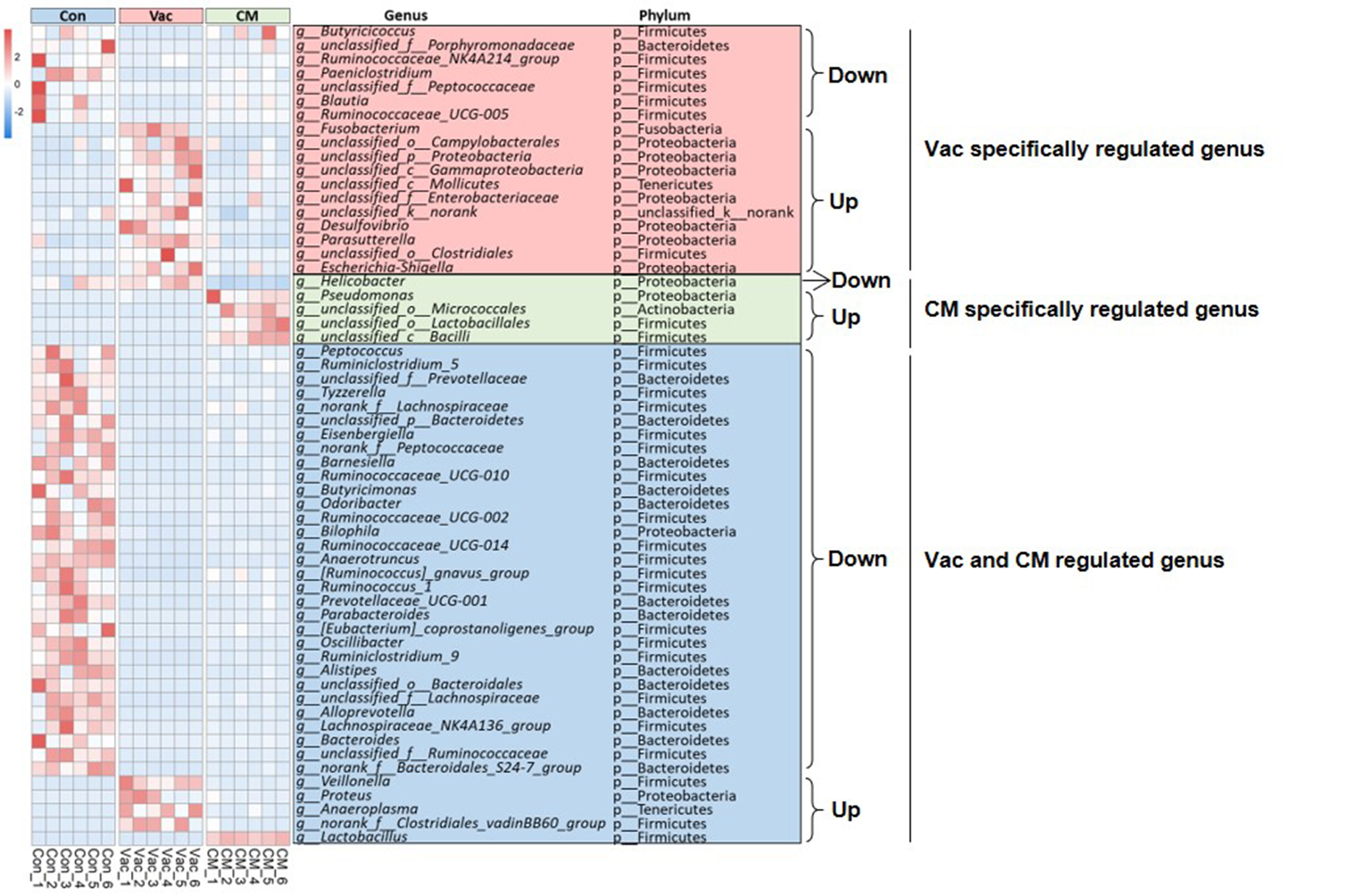

Supplement: Supplementary Figure 1 — Differential bacteria between antibiotic intervention group and control group at genus level (p < 0.01 under Mann–Whitney U test). [file Image_1.jpg]

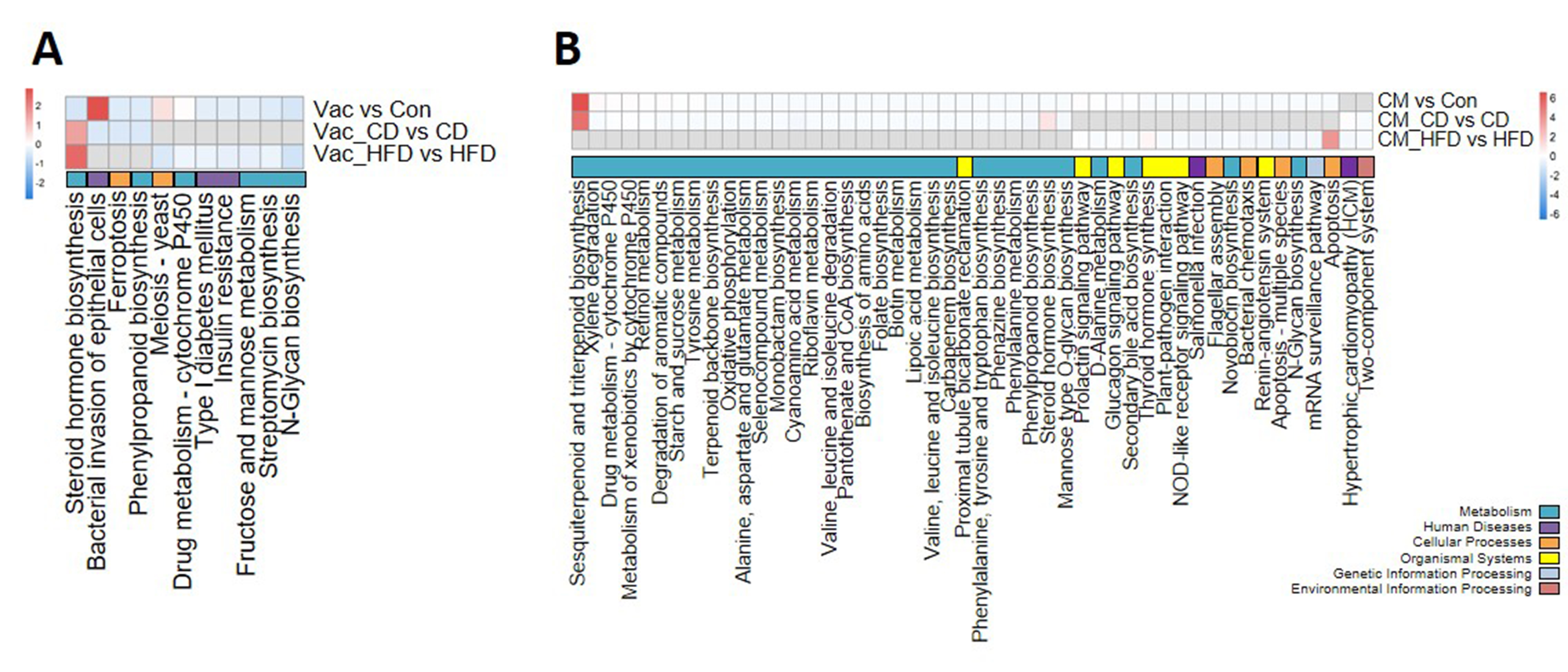

Supplement: Supplementary Figure 5 — Shared bacterial functional pathways among three conditions. (A) The 11 shared pathways between Vac vs. Con, Vac_CD vs. CD and Vac_HFD vs. HFD on KEGG pathway level 3 were shown with heatmap (Log2 Fold change). (B) The 48 shared pathways between CM vs. Con, CM_CD vs. CD and CM_HFD vs. HFD on KEGG pathway level 3 were shown with heatmap (Log2 Fold change). [file Image_5.jpg]
