# Supplementary material for: Integrated Metagenomic and Transcriptomic Analyses Reveal the Dietary Dependent Recovery of Host Metabolism From Antibiotic Exposure
Source: Front Cell Dev Biol. 2021 Jun 18;9:680174. doi: 10.3389/fcell.2021.680174 (PMC8250461; doi:10.3389/fcell.2021.680174)
Supplement: Supplementary file 6 [file Table_1.DOCX]

**Supplemental Table 1.** Differential pathways between Vac-treated and control groups that are commonly appeared after 5-day Vac intervention and after 45-day recovery on either CD or HFD.

|  | **Antibiotic intervention** | | **Recovery on CD** | | **Recovery on HFD** | |
| --- | --- | --- | --- | --- | --- | --- |
| **Pathway level 1** | **Pathway level 3** | ***p* value** | **Pathway level 3** | ***p* value** | **Pathway level 3** | ***p* value** |
| Metabolism | Glutathione metabolism | 0.000 | Glutathione metabolism | 0.012 | Glutathione metabolism | 0.044 |
| Cellular Processes | Cell cycle | 0.015 |  |  | Cell cycle | 0.002 |
| Cellular Processes | p53 signaling pathway | 0.022 |  |  | p53 signaling pathway | 0.002 |
| Environmental Information Processing | Cytokine-cytokine receptor interaction | 0.017 |  |  | Cytokine-cytokine receptor interaction | 0.022 |
| Human Diseases | Measles | 0.011 |  |  | Measles | 0.004 |
| Human Diseases | Maturity onset diabetes of the young | 0.014 |  |  | Maturity onset diabetes of the young | 0.030 |
| Organismal Systems | Prolactin signaling pathway | 0.031 |  |  | Prolactin signaling pathway | 0.002 |
| Human Diseases |  |  | Autoimmune thyroid disease | 0.037 | Autoimmune thyroid disease | 0.000 |
| Human Diseases |  |  | Leishmaniasis | 0.037 | Leishmaniasis | 0.000 |
| Human Diseases |  |  | Allograft rejection | 0.033 | Allograft rejection | 0.000 |
| Human Diseases |  |  | Staphylococcus aureus infection | 0.011 | Staphylococcus aureus infection | 0.000 |
| Organismal Systems |  |  | IL-17 signaling pathway | 0.029 | IL-17 signaling pathway | 0.001 |
