# Supplementary material for: Integrated Metagenomic and Transcriptomic Analyses Reveal the Dietary Dependent Recovery of Host Metabolism From Antibiotic Exposure
Source: Front Cell Dev Biol. 2021 Jun 18;9:680174. doi: 10.3389/fcell.2021.680174 (PMC8250461; doi:10.3389/fcell.2021.680174)
Supplement: Supplementary file 7 [file Table_2.DOCX]

**Supplemental Table 2.** Differential pathways between CM-treated and control groups that are commonly appeared after 5-day CM intervention and after 45-day recovery on either CD or HFD.

|  | **Antibiotic intervention** | | **Recovery on CD** | | **Recovery on HFD** | |
| --- | --- | --- | --- | --- | --- | --- |
| **Pathway level 1** | **Pathway level 3** | ***p* value** | **Pathway level 3** | ***p* value** | **Pathway level 3** | ***p* value** |
| Metabolism | Metabolism of xenobiotics by cytochrome P450 | 0.034 | Metabolism of xenobiotics by cytochrome P450 | 0.011 |  |  |
| Human Diseases | African trypanosomiasis | 0.000 |  |  | African trypanosomiasis | 0.044 |
| Human Diseases | Amoebiasis | 0.012 |  |  | Amoebiasis | 0.048 |
| Organismal Systems | Adipocytokine signaling pathway | 0.047 |  |  | Adipocytokine signaling pathway | 0.024 |
| Environmental Information Processing |  |  | Cytokine-cytokine receptor interaction | 0.036 | Cytokine-cytokine receptor interaction | 0.024 |
